# Preservation of nasal turbinates in endoscopic, anterior skull base surgery—yes, we can!

**DOI:** 10.1007/s00405-021-06856-9

**Published:** 2021-05-08

**Authors:** Axel Wolf, Alexandros Andrianakis, Peter Valentin Tomazic, Michael Mokry, Georg Clarici, Etienne Holl, Thomas Weiland, Peter Kiss, Sarah Vasicek, Anna Brunner, Christian Lehner, Johannes Schwarz, Verena Gellner

**Affiliations:** 1 Department of Otorhinolaryngology, Head and Neck Surgery, Medical University of Graz, Graz, Austria; 2 Department of Neurosurgery, Medical University of Graz, Graz, Austria

**Keywords:** Anterior skull base, Endoscopic skull base surgery, Skull base neoplasms, Nasal turbinates, Preservation

## Abstract

**Objective:**

To evaluate the frequency, type and indications of nasal turbinate (NT) resection during endoscopic, anterior skull base surgery and to analyze factors that may have an impact on the need of NT removal.

**Methods:**

In this retrospective cohort study, 306 subjects (150 males and 156 females, mean age 55.4 ± 15.3 years) who underwent multidisciplinary, transnasal, endoscopic tumor surgery of the anterior skull base using 4-handed techniques between 2011 and 2019 at the Department of Otorhinolaryngology, Medical University of Graz, were included.

**Results:**

In the majority of interventions (*n* = 281/306; 91.8%), all NT were preserved. Significant factors influencing the need of NT resections turned out to be type of endoscopic approach (*p* < 0.001; V = 0.304), sagittal (*p* = 0.003; d = 0.481) and transversal (*p* = 0.017; d = 0.533) tumor diameter, tumor type (*p* < 0.001; *V* = 0.355) and tumor location (*p* < 0.001; *V* = 0.324).

**Conclusions:**

NT can be preserved in the majority of patients undergoing tumor resection in anterior, transnasal, skullbase surgery and routine resection of NT should be avoided. Variables that have an impact on the need of NT resections are types of endoscopic approaches, sagittal and transversal tumor extension and tumor type. These factors should be considered in planning of surgery and preoperative information of patients.

## Introduction

Nasal turbinates (NTs) are bony lamellas originating from the lateral nasal wall covered by respiratory and olfactory epithelium. In the human nose, inferior, middle, superior and sporadically supreme turbinates can be found. NTs increase the surface area of the nose (up to 200 cm^2^) which improves the warming and the humidification of the inspiratory air and the cooling of the expiratory air. Furthermore, turbinates provide the filtration of the nose by secreting a mucous, which traps small particles between 3 und 0.5 µm and contains various antibodies playing a vital role in the immune system of the nose [1, 2]. In addition, olfactory neuroepithelium can be present on the superior as well as on the middle turbinate—as part of the olfactory system [3].

Endoscopic approaches to the nose and paranasal sinuses were introduced in the 1980s. According to surgical principles of Messerklinger and Stammberger school, physiologic and healthy structures of the nose have to be respected and preserved in functional, endoscopic, transnasal surgery [4]. After decades of experience and technical advances (curved drills, angled endoscopes, etc.) indications of the technique have extended tremendously. Extended endoscopic approaches to the anterior skull base (SB) are used in clinical routine today. Although indications for endonasal, endoscopic sinus surgery have become broader and broader, a low morbidity and mortality rate have to be considered during surgical procedures.

In endoscopic surgery of the SB, sufficient visualization of the surgical field has to be provided to perform save procedures [5]. Thus, approaches have to be chosen according to tumor localization, extension and anatomic situations according to the individual anatomy of patients. Furthermore, enough space for the use of defect-closure techniques (i.e. nasoseptal flaps) must be provided, if needed [6]. During the creation of an approach to the SB, physiologic structures such as nasal turbinates should be preserved, if possible [7]. In particular, the middle and superior turbinates are mostly manipulated in order to provide a sufficient corridor for surgical manipulation at the SB.

Although effects of the resection of nasal turbinates has been controversially discussed, resection of the nasal turbinates can lead to chronic dysfunction of the nasal physiology: reduced sense of smell, nasal dryness, impaired frontal sinus drainage, recurrent epistaxis, crusting and the empty nose syndrome are potential complications after removal of nasal turbinates. The empty nose syndrome is caused by a turbinectomy of the inferior and/or middle tubinates theoretically. It is mainly characterized by the sensation of a paradox obstruction of the nasal airway. It also leads to feelings of nasal dryness and dyspnea, hyperventilation and is even associated with psychiatric illnesses. Therefore, empty nose syndrome can lead to substantial quality of life impairments [8–11]. To maintain physiologic function of the nose it is our concept to avoid resection of nasal turbinates in endoscopic SB surgery whenever possible.

The aim of this study is to evaluate the frequency, type and indications of NT resections during surgical resections of tumors of the anterior SB at an academic center with more than two decades of experiences in multidisciplinary endoscopic skull base surgery. Furthermore, we wanted to analyze factors (tumor type, tumor size, tumor location, surgical approach, pneumatisation of the sphenoidal sinus and SB defect closure techniques) that may have an impact on the need of turbinate removal to provide better preoperative planning and to avoid (over-) resection of NTs during surgery.

## Materials and methods

### Subjects

This retrospective cohort study was carried out at the Medical University of Graz, Department of Otorhinolaryngology, Head and Neck Surgery, in collaboration with the Department of Neurosurgery. All patients were identified through the institutional patient registry with tumors of the anterior SB, who were treated by multidisciplinary, transnasal, endoscopic surgery between 2011 and 2019. Diagnosis was determined by clinical, radiologic (computed tomography [CT] and magnet resonance tomography [MRT]), blood (including hormonal testing) and histologic examination. Indication for transnasal, endoscopic approaches was given through the institutional, multidisciplinary SB board.

### Clinical parameters

Patients’ charts were retrospectively reviewed with respect to demographic data, removal of nasal turbinates, tumor entity, tumor localization, tumor size, surgical approach, pneumatization of the sphenoid sinus and SB defect closure techniques. Tumor size was measured in the maximal diameters of the craniocaudal, sagittal and transversal axes. Tumor location was categorized in sellar, sellar with extrasellar extensions and extrasellar (“extrasellar” locations included sinus cavernosus, sinus sphenoidalis, orbita, nasal cavity, clivus, para- and suprasellar area, ethmoid, cribriform plate). The pneumatization of the sinus sphenoidalis was classified into conchal, presellar and sellar types [6].

### Surgical procedures

All patients underwent multidisciplinary (neurosurgery and otorhinolaryngology) transnasal endoscopic surgery from using 4-handed techniques. For all endoscopic approaches, various endoscopes (0, 45, and 70 angles of view), a broad range of surgical instruments (including standard and diamond drills, microdebriders, and cold steel instruments) as well as intraoperative navigational systems have been available. During procedures, topical vasoconstriction (adrenalin 1:1000) was applied. If needed, corrections of significant septal deviations were performed. Furthermore, lateralization of middle turbinates was performed and resections of the posterior nasal septum and sphenoidal septum was performed in transsphenoidal approaches for two-nostril approaches. Surgical approaches were classified as into transsellar, transtubercular and other approaches (transorbital, transclival, transsphenoidal, transcribriform, transethmoidal). The removal of the turbinates was classified into the concha types (inferior, middle, superior), total and subtotal resection. Furthermore, the use of nasoseptal (‘Hadad’) flaps and fascia lata for closure of SB defects was evaluated.

### Statitical analysis

SPSS^©^ statistical software, version 25.0 (IBM^©^, Armonk, NY) was used for statistical analysis. Continuous variables are presented as means ± standard deviation and categorical variables as absolute numbers and percentages. For comparison of continuous variables, unpaired t-tests were utilized. In presence of variance inhomogeneity, robust Welch’s *t* test was used. *t* test results were presented with the mean difference (M^diff^) and 95% confidence interval (CI). The effect size of statistically significant differences in unpaired t-tests was expressed by Cohen’s *d*. For comparison of categorical variables, Chi-squared test was performed. Results of Chi-squared test were presented with the difference in frequency distribution (*D*). Cramer’s *V* coefficient was used to evaluate the effect size of the overall statistically significant difference in Chi-squared analysis. Post-hoc *Z* tests in Chi-squared analysis were conducted with Bonferroni adjustment in order to correct multiplicity. Effect size of statistically significant post-hoc *Z* tests was determined with the φ-coefficient. Type of power analysis was post-hoc—given α, sample size and effect size. All statistical tests were two-sided and statistically significant α level was set at *p* < 0.05.

### Ethical considerations

The study was approved by the institution’s local Ethics Committee (EK-Nr. 31-431 ex 18/19) and conducted according to the declaration of Helsinki on Biomedical Research Involving Human Subjects. Due to the retrospective nature of this study, requirement of patient’s informed consent was waived by the local ethics committee. Clinical records were anonymized prior to analysis.

## Results

### Total cohort analysis

306 subjects [150 males (49.1%) and 156 females (50.1%), mean age 55.4 ± 15.3 years] were included in this retrospective investigation.

Tumor entities were distributed as follows: macroadenomas were found in 202 (66%), meningiomas in 16 (5.2%), craniopharyngiomas in 15 (4.9%), microadenomas in 13 (4.2%), Rathke's cleft cysts in 9 (2.9%) and chordomas in 7 (2.3%) cases. Other lesions were observed in 44 (14%) patients. Regarding tumor locations, 115 tumors (37.6%) were located solely in the sellar area, 134 sellar tumors (43.8%) extended into the surrounding regions and 57 tumors (18.6%) were found outside the sellar area. Due to limited availability of radiologic imaging, tumor dimensions could not be evaluated in all patients. Sagittal and transversal diameters were evaluated in 299 patients and craniocaudal diameter was evaluated in 72 patients. Mean sagittal, transversal and craniocaudal tumor diameter were 1.9 ± 0.8, 2.1 ± 0.8 and 2.3 ± 1.1 cm, respectively.

Type of surgical approach could be evaluated in 290 patients, as in 16 cases the surgical report was insufficiently documented. In almost all cases, a binostril surgical approach was applied (*n* = 285/290, 98%). Transsellar, transtubercular and other approaches were performed in 237 (81.7%), 18 (6.2%) and 35 (12.1%) subjects, respectively. Performed “other approaches” included transsphenoidal (*n* = 12, 4.1%), transclival (*n* = 8, 2.7%), transethmoidal (*n* = 8, 2.7%), transorbital (*n* = 4, 1.4%) and transcribriform (*n* = 3, 1%). In 45 patients (14.7%), closure of anterior SB defect by Nasoseptal (‘Hadad’) flap and fascia lata was recorded. Again, due to limited availability of radiologic imaging, pneumatization of the sphenoid sinus could be assessed in 269 patients. Conchal, presellar and sellar types were found in 11 (4%), 54 (20%) and 204 (76%) subjects, respectively.

In 15 (4.9%) patients, one turbinate was resected and in 9 (2.9%) patients, two turbinates were removed. In total, 33 turbinates have been partially (*n* = 7, 21.2%) or totally (*n* = 26, 78.8%) resected. Most commonly the middle (*n* = 17, 51.5%) and superior (*n* = 15, 45%) turbinates were resected, with one case (3%) of inferior turbinate removal. In addition, in one patient, surgical report did describe a turbinate resection but did not specify the extent or type of the procedure. To summarize, in 25 out of 306 (8.2%) patients a turbinate resection was warranted during surgery. In 19 (76%) out of these subjects, a specific reason for this measure was described by the surgeon: small nasal cavity (*n* = 5, 26%), enlarged (i.e. concha bullosa) and/or rigid turbinates (*n* = 4, 21%), anatomic proximity to tumor localization (*n* = 3, 15.8%), decalcefied turbinates (*n* = 2, 10.5%), and one case (5.2%) of a low pneumatized sphenoid sinus. In the remaining 6 (24%) subjects, no specific indication for turbinate removal was described within surgical reports. In similarity to the total cohort, a binostril surgical approach was performed in the vast majority of turbinate resection cases (n = 23/25, 92%).

### Group comparisons

Group comparisons between the patient group without turbinate resection [*n* = 281 subjects, 142 (50.5%) males and 139 (49.5%) females, mean age 55.6 ± 17.1 years] and the turbinate resection group [*n* = 25 subjects, 14 (56%) males and 11 (44%) females, mean age 54.34 ± 17.94 years] were performed. There was no statistically significant difference in sex distribution [χ^2^(1) = 0.27, *p* = 0.600, *D* = 1%] and mean age [*t*(304) = 0.36, *p* = 0.719, M^diff^ = 1.3, 95%CI = −5.7 to 8.3] between groups.

Chi-squared analyses showed a significant impact of tumor type on the removal of nasal turbinates [χ^2^(6) = 38.50, *p* < 0.001, *V* = 0.355, power = 99%]. Meningiomas had the highest rate (37.5%) of turbinate resection within tumor types. Detailed results of post-hoc tests are displayed in Table 1. Furthermore, there was a statistically significant difference between groups in distribution of tumor location [χ^2^ (2) = 32.22, *p* < 0.001, *V* = 0.324, power = 99%]. According to post-hoc results, extrasellar tumors had a statistically significant higher rate of turbinate resection compared to tumors solely located in the sellar area (D = 24.6%, *p* < 0.001, *φ* = 0.387, power = 99%) and sellar tumors with extension into the surrounding regions (*D* = 20.3%, *p* = 0.001, *φ* = 0.286, power = 98%). Detailed post-hoc data is given in Table 2. Extrasellar tumors with need of turbinate resection (*n* = 15) were located at the planum sphenoidale (*n* = 4), sphenoid wings (*n* = 4), ethmoid (*n* = 2), clivus (*n* = 2), fossa pterygopalatine (*n* = 1), cribriform plate (*n* = 1) and orbit (*n* = 1).

**Table 1:** Impact of tumor entity on turbinate resection

Tumor entity	Turbinate resection (*n* = 25)	No turbinate resection (*n* = 281)	*p* value
Macroadenoma, *n* = 202	7 (3.5)	195 (96.5)	< 0.001*
Microadenoma, *n* = 13	0 (0)	13 (100)	0.271
Meningioma, *n* = 16	6 (37.5)	10 (62.5)	< 0.001*
Rathke’s cleft cyst, *n* = 9	0 (0)	9 (100)	0.363
Chordoma, *n* = 7	0 (0	7 (100)	0.424
Craniopharyngioma, *n* = 15	3 (20)	12 (80)	0.086
Others, *n* = 44	9 (20.5)	35 (79.5)	0.001*

Values are presented in absolute numbers and percentages, *n* (%)

*Represents statistically significance at the Bonferroni adjusted α-level (0.05/14 = 0.003)

**Table 2:** Impact of tumor location on turbinate resection

Tumor location	Turbinate resection (*n* = 25)	No turbinate resection (*n* = 281)	*p* value
Sellar, *n* = 115	2 (1.7)	113 (98.3)	0.214
Sellar with extrasellar extension, *n* = 134	8 (6)	126 (94)	0.001*
Extrasellar, *n* = 57	15 (26.3)	42 (73.7)	< 0.001*

Values are presented in absolute numbers and percentages, *n* (%)

*Represents statistically significance at the Bonferroni adjusted α-level (0.05/6 = 0.008)

In terms of tumor dimensions, a statistically significant difference between turbinate resection groups was found in transversal [*t *(25.6) = 2.55, *p* = 0.017, *d* = 0.533, power = 81%] and sagittal [*t *(297) = 2.99, *p* = 0.003, *d* = 0.481, power = 74%] diameter. Patients with turbinate resection had a significantly higher transversal [M^diff^ = 0.7 cm, 95%CI = 0.1–1.2] and sagittal [M^diff^ = 0.5 cm, 95%CI = 0.1–1.1] diameter than patients without turbinate removal. Craniocaudal diameter did not differ statistically significant between groups [*t* (70) = 1.67, *p* = 0.099, M^diff^ = 0.6 cm, 95%CI = 0.1–1.3]. Remaining data of tumor dimensions according to turbinate resection groups are depicted in Table 3.

**Table 3:** Impact of tumor size on turbinate resection

Tumor diameter	Turbinate resection	No turbinate resection	*p* value
Transversal, *n* = 299	2.8 ± 1.3 *n* = 25	2.1 ± 0.8 *n* = 274	0.017*
Sagittal, *n* = 299	2.4 ± 1.2 *n* = 25	1.8 ± 0.7 *n* = 274	0.003*
Craniocaudal, *n* = 72	2.9 ± 1.2 *n* = 8	2.3 ± 1 *n* = 64	0.099

Values are presented in means ± standard deviations in cm

*Represents statistically significance at the 0.05 level

Furthermore, a statistically significant difference between turbinate resection groups was found in distribution of endoscopic approach types [χ^2^(2) = 26.76, *p* < 0.001, *V* = 0.304, power = 99%]. Post-hoc analysis revealed that transsellar approaches showed a significant lower rate of turbinate resection compared to transtubercular (*D* = 18.4%, *p* < 0.001, *φ* = 0.214, power = 83%) and other approaches (*D* = 21.9%, *p* < 0.001, *φ* = 0.295, power = 98%), see Table 4. Due to the latter significance, turbinate resection was further analyzed for each individual approach type within the “other approach group”: Transethmoidal approach had the highest resection rate (*n* = 4/8; 50%), followed by transcribriform (*n* = 1/3; 33.3%), transorbital (*n* = 1/4; 25%), transclival (*n* = 2/8; 25%) and transsphenoidal (*n* = 1/12; 8.3%).

**Table 4:** Impact of surgical approach on turbinate resection

Surgical approach	Turbinate resection (*n* = 22)	No turbinate resection (*n* = 268)	*p*-value
Transsellar, *n* = 237	9 (3.8)	228 (96.2)	< 0.001*
Transtubercular, *n* = 18	4 (22.2)	14 (77.8)	0.015
Other approaches, *n* = 35	9 (25.7)	26 (74.3)	< 0.001*

Values are presented in absolute numbers and percentages, *n* (%)

*Represents statistically significance at the Bonferroni adjusted α-level (0.05/6 = 0.008)

Our analysis regarding sphenoid pneumatization showed that there were no statistically significant differences in turbinate resection rate [χ^2^(2) = 6.21, *p* = 0.054] between conchal types (*n* = 3/11, 27.3%), presellar types (*n* = 3/54, 5.6%) and sellar types (*n* = 15/204, 7.4%). Furthermore, no statistically significant difference in turbinate removal prevalence [χ^2^(1) = 3.84, *p* = 0.071, *D* = 8.7%] was found between cases with an anterior SB defect closure by nasoseptal (‘Hadad’) flaps/fascia lata (*n* = 7/45, 15.6%) and without (*n* = 18/261, 6.9%).

## Discussion

In this study, we evaluated frequency, type, indications and influencing factors of nasal turbinate resection in 306 patients undergoing multidisciplinary, transnasal endoscopic tumor surgery of the anterior SB using 4-hand techniques. Overall, we could show that in the majority of all interventions (*n* = 281/306; 91.8%) all nasal turbinates were preserved. Statistical analysis revealed, that factors influencing the need of turbinate resections are type of endoscopic approach, tumor entity, -dimension and -location. To the best of our knowledge, this is the first clinical study that focuses on factors that may influence turbinate resection during endoscopic resection of tumors of the anterior SB.

Since possibilities of endoscopic surgery have extended, a lot or procedures can be done beyond limitations of the past. While many surgeons describe the routine sacrifice one or both middle turbinates in transsphenoidal SB surgery [5, 12–14], other surgeons advocate turbinate-sparing techniques, following principles of endoscopic transnasal surgery of the Messerklinger and Stammberger school [4, 11].

On one hand, reported benefits of turbinate resection comprise better increased exposure of the SB, increased postoperative sinunasal patency and the use of free mucosal grafts from the middle turbinate [5, 12–15]. On the other hand, turbinate sparing surgery aims to preserve the physiologic function of the nose and avoids complications associated with turbinectomy. In general, complications of turbinate removal include reduced sense of smell, empty nose syndrome, increased nasal dryness, development of frontal sinusitis, increased risk of epistaxis and increased crusting postoperatively. Furthermore, loss of anatomical landmarks can be challenging in revision surgery [9–11, 16, 17]. Thompson et al. showed that nasal symptoms improved faster in patients without turbinate resection after surgery [17]. Resection of the middle and lower nasal turbinate is, in general, a likely risk factor for the occurrence of empty nose syndrome [18]. Maza et al. showed, that patients with resections of superior and middle turbinates during transnasal endoscopic surgery of the anterior skull base may suffer from similar symptoms to patients with inferior turbinectomy [9]. In addition, resection of the middle and inferior turbinates may cause hyposomnia due to alteration in nasal airflow, although resection of the middle nasal turbinate as a cause of olfactory loss is discussed controversially in literature [19, 20].

Based on literature and experience, it is our concept to maintain physiologic structures of the nose in endoscopic skull base surgery, whenever possible. In the present study, we could show that in 91.8% of patients, all nasal turbinates were preserved and sufficient access to lesions at the SB were provided. In particular, most transsellar approaches (96.3%) can be applied without turbinate resection in our experience. In consistency with Nyquist et al. [11], who reported a very high rate (98%) of middle turbinate preservation in endoscopic transsphenoidal surgery of the anterior SB, we could show that the preservation of physiologic structures of the nose is possible in majority of patients.

As a next step, we aimed to evaluate factors that may have had an impact on the need of turbinate resection during SB surgery in order to plan surgical procedures more detailed, to avoid routine (over-) resection nasal turbinates and to preoperative information of patients. Group comparisons (turbinate-resection and no-turbinate resection) revealed, that enlarged tumor size in its sagittal and transversal maximal diameter is a risk factor for the need of turbinate resection. We assume, that larger tumors are more likely to protrude into critical anatomic regions. Thus, in particular the maximum transverse diameter might play an important role: in the case of an extensive lateral extension, turbinates may represent an additional obstacle. This theory is supported by the fact that the largest deviation in size between the resection and non-resection group can be found in this axis. No statistically significant difference was found for the maximal craniocaudal diameter. This might be explained due to the lack of data of cases in which the craniocaudal diameter could be measured in this retrospective setting. Since there is statistical significance for two of the three axes, it may be assumed that the resection of turbinates is connected to the overall size of the lesions.

As expected, Macroadenomas of the pituitary gland showed the lowest turbinate resection rates compared to other entities. This, of course, can be explained due to the increased occurrence of certain types of tumors at specific sites, but also due to different surgical aims. For example, malignant tumors require more radical surgery than adenomas and thus require turbinectomies more frequently.

A significant difference in turbinate resection rate was found between the various surgical approaches. A transsellar approach showed by far the lowest rate of turbinate resection. This result is in accordance with the tumor entity data, as transsellar approach is used for removal of macroadenomas of the pituitary gland. Thus, the surgical window in case of a transsellar approach provides enough space to preserve the nasal turbinates. In contrast, the need of turbinate resection was significant higher in the “other approach group”. A further analysis revealed that transethmoidal and transcribriform approaches showed the considerably highest turbinate resection rates. Transethmoidal approach was performed to either excise tumors situated ethmoidal or to sufficiently reach tumors located at the far lateral parts of the sphenoid. In such approaches, turbinate resection is often not to avoid. Similar applies for transcribriform approaches, which were performed to remove tumors located at the cribriform plate.

Related to this point, analysis of tumor locations showed, that extrasellar tumors had a significant higher rate of turbinate resection compared to tumors solely located in the sellar area and sellar tumors with extension into the surrounding regions. Thus, tumors originating from the sella region showed the lowest rate of turbinate resection. In accordance to analysis of tumor entities and surgical approaches, these locations are typical for adenomas of the pituitary gland and can be reached by a transsellar approach [21, 22]. Contrary, extrasellar tumors often requires a broader surgical window to be excised totally, hence, turbinate resection for this purpose may be necessary in these cases.

It is important to underline that factors such as tumor type, -location, -size and surgical approach are closely intertwined and should be seen as a combination of factors.

Analysis of pneumatization of the sphenoid sinus showed the trend, that low pneumatization (conchal type) might harbor a higher risk of turbinate resection compared to sellar and presellar types. The lack of statistical significance might be explained due to the limited size of the cohort. It is important to underline, that even in the majority of conchal-type cases, all nasal turbinates have been preserved, thus, low sphenoid sinus pneumatization is no obligatory indication for nasal turbinate resection.

For further analysis, we decided to describe surgeons’ subjective parameters as indication for turbinate resection intraoperatively: narrow anatomy of the nasal cavity was described in most of the cases. Furthermore, the individual configuration of nasal turbinates (concha bullosa/rigid turbinates) seem to be indications for turbinate resection in certain cases. As expected, anatomic proximity of lesions and surgical fields have been described. Taken together, a number of different, individual, anatomic variations seem to harbor risks for the need of turbinate resection. Thus, turbinate resections should be indicated according to the individual intraoperative situations.

In addition, we investigated the factors age and gender to determine their influence on the resection of turbinates. Results showed, that neither a significant difference in the frequency of turbinate resection between men and women nor an association between the patients’ age and nasal turbinate resection was found.

No significant association between the use of nasoseptal (‘Hadad’) for closure of SB defects and the removal of a nasal turbinates was shown. In the majority of cases, in which ‘Hadad’-flaps were applied, all nasal turbinates have been preserved. In contrast to our experience, in literature, routine resection of the middle turbinate while using nasoseptal flaps is described [23, 24]. Thus, our findings support a conservative approach to turbinate resections if nasoseptal flaps are used.

Although a main limitation of this study is its retrospective design with a lack of evaluation of postoperative nasal outcome in our cohort, we could show that resection of nasal turbinates is not obligatory to provide sufficient visualization or to provide a sufficient working-corridor in endoscopic SB surgery. Furthermore, not all CT- and MRT scans have been available in different planes: Thus, evaluation of tumor-dimension as well as pneumatization of the sphenoid sinus was not possible in all cases.

## Conclusion

In the present study, we showed that nasal turbinates can be preserved in the majority of cases in endoscopic anterior SB surgery. Based on the results of the present study, routine resection of nasal turbinates in endoscopic anterior SB surgery should be avoided. Extensive, and technically challenging endoscopic procedures can be performed in 4-hand techniques without resection of nasal turbinates. If needed for safe and total completion of surgical aims, turbinate resection should be indicated carefully to preserve physiological nasal function. Variables that have an impact on the need of turbinate resections are type of endoscopic approach, tumor dimension, -location and -entity. Thus, these factors should be considered in preoperative planning of surgery and preoperative informed consent of patients.

## Data Availability

The datasets generated and analyzed during the current study are available from the corresponding author on reasonable request.
